# B Cell-Derived Extracellular Vesicles Reveal Residual B Cell Activity in Kidney Graft Recipients Undergoing Pre-Transplant Desensitization

**DOI:** 10.3389/fmed.2021.781239

**Published:** 2021-12-16

**Authors:** David Cucchiari, Valeria Tubita, Jordi Rovira, Maria J. Ramirez-Bajo, Elisenda Banon-Maneus, Marta Lazo-Rodriguez, Natalia Hierro-Garcia, Francesc E. Borràs, Pedro Ventura-Aguiar, Gastón J. Piñeiro, Jaume Martorell, Lluís Peri, Mireia Musquera, Alexandre Hertig, Federico Oppenheimer, Josep M. Campistol, Fritz Diekmann, Ignacio Revuelta

**Affiliations:** ^1^Department of Nephrology and Kidney Transplantation, Hospital Clínic, Barcelona, Spain; ^2^Laboratori Experimental de Nefrologia i Trasplantament (LENIT), Institut d'Investigacions Biomèdiques August Pi i Sunyer (IDIBAPS), Barcelona, Spain; ^3^Red de Investigación Renal (REDINREN), Madrid, Spain; ^4^REMAR-IVECAT Group, “Germans Trias i Pujol” Health Science Research Institute, Badalona, Spain; ^5^Department of Cell Biology, Physiology and Immunology, Universitat Autònoma de Barcelona, Cerdanyola del Vallès, Spain; ^6^Department of Immunology, Hospital Clínic, Barcelona, Spain; ^7^Department of Urology, Hospital Clínic, Barcelona, Spain; ^8^Service de Néphrologie, Hôpital Foch, Suresnes, France

**Keywords:** B cells, kidney transplantation, desensitization, HLA-incompatibility, extracellular vesicles (EV), exosomes, plasma cells

## Abstract

**Background:** Living-donor kidney transplant (LDKT) recipients undergoing desensitization for Human Leukocyte Antigen (HLA)-incompatibility have a high risk of developing antibody-mediated rejection (ABMR). The purpose of the study is to evaluate if residual B cell activity after desensitization could be estimated by the presence of circulating B cell-derived extracellular vesicles (BEVs).

**Methods:** BEVs were isolated by Sepharose-based size exclusion chromatography and defined as CD19+ and HLA-II+ extracellular vesicles. We analyzed stored serum samples from positive crossmatch LDKT recipients before and after desensitization at first post-transplant biopsy and at 12-month protocol biopsy (*n* = 11). Control groups were formed by hypersensitized patients who were not submitted to desensitization (*n* = 10) and by low-risk recipients (*n* = 9). A prospective validation cohort of 11 patients also included the analysis of B cells subpopulations in recipients' blood and lymph nodes recovered upon graft implantation, along with BEVs analysis before and after desensitization.

**Results:** We found out that CD19+ and HLA-II+BEVs dropped significantly after desensitization and relapse in patients who later developed ABMR was evident. We validated these findings in a proof-of-concept prospective cohort of 6 patients who received the same desensitization protocol and also in a control group of 5 LDKT recipients. In these patients, B cell subpopulations were also studied in recipients' blood and lymph nodes that were recovered before the graft implantation. We confirmed the significant drop in BEVs after desensitization and that this paralleled the reduction in CD19+cells in lymph nodes, while in peripheral blood B cells, this change was almost undetectable.

**Conclusions:** BEVs reflected B cell residual activity after desensitization and this could be a valid surrogate of humoral alloreactivity in this setting.

## Introduction

Patients with chronic kidney disease that are sensitized to HLA antigens have limited access to kidney transplantation, resulting to increased mortality in the waiting list. A possibility to overcome the HLA barrier is represented by desensitization before kidney transplantation. This regimen, which is usually based on plasma exchange, intravenous immunoglobulins, and anti-CD20 antibodies, is being done in order to reduce the quantity of circulating donor-specific antibodies (DSAs) and to deplete B cells (1–3). This option is associated with favorable results, as graft and patient survival are undoubtedly better when compared to hypersensitized patients on dialysis waiting for a compatible donor (4). However, despite the reasonable solid outcomes, incidence of antibody-mediated rejection (ABMR) is as high as 30–50% (5–7), and forces a transplant physician to enhance immunosuppression but with concerns of higher rate of infectious, neoplastic and cardiovascular complications (7, 8).

The effects of desensitization on DSA titer can be controlled using solid-phase techniques. However, how B cell biology is affected by this treatment remains unclear. One possibility is to think that circulating B cells represented a good estimate of treatment efficacy. However, while circulating B cells are easily depleted after a single dose of anti-CD20 antibody, a high proportion of B cells survived in lymph nodes with a switched-memory phenotype (9). To check activity and proliferation of residual B cells after desensitization, a fine-needle aspiration of bone marrow or lymph node biopsy is unpractical, apart from being invasive. Therefore, we hypothesized that the activity of B cells, which survived in lymphoid organs after desensitization, can be estimated by the presence of circulating B cell-derived extracellular vesicles (BEVs).

Extracellular Vesicles (EVs) are a heterogeneous population of cell-derived vesicles of various origins and sizes, including exosomes, microvesicles, and apoptotic bodies (10). They played a key role in intercellular communication, delivering signal molecules (proteins, nucleic acids, lipids, etc.) that can regulate immune functions (11). Concretely, EVs derived from immune cells are implicated in antigen presentation, immunoregulation, and viral transmission (12, 13). B cells actively secrete EVs upon proliferation stimuli, such as T-cell help via CD40 and IL-4 signaling (11, 13). Importantly, BEVs display markers of B cell (CD19, IgM, IgG) and of antigen-presenting cells origin (HLA-I, HLA-II, CD86) (11).

The possibility of circulating EVs to act as efficient biomarkers has been recently highlighted in different fields. In oncology, for example, tumor-derived circulating exosomes are associated with the burden of the primary mass (14). In kidney transplantation, mRNA transcripts from circulating exosomes are associated with rejection phenotypes (15). Our group recently highlighted the importance of transplant immunosuppression in EV content from colorectal cancer cell lines in the regulation of the pre-metastatic niche (16). It is reasonable to speculate that circulating BEVs reflect B cell proliferation in bone marrow and lymphoid organs, even though the circulating B cells are not detectable because of desensitization. To prove this hypothesis, we developed a retrospective and a prospective study in living-donor kidney transplant (LDKT) recipients who were undergoing a desensitization protocol based on anti-CD20 antibodies, plasma exchanges, and intravenous immunoglobulins.

## Materials and Methods

### Study Population

The retrospective cohort included patients who had kidney transplantation procedure across a 10-year period (2006–2015). Demographics and clinical characteristics of both donors and recipients have been collected, along with immunological profiles of recipients, HLA-matching, and immunosuppressive treatment. Patients were divided into three groups according to the immunological risk: patients who received desensitization before transplantation for a positive crossmatch (“DS” group, *n* = 11), hypersensitized patients with a cPRA I+II > 85% not submitted to desensitization and without a DSA (“HS” group, *n* = 10), and a low-risk group with baseline cPRA I+II <10% and no DSAs (“CT” group, *n* = 9). For those patients who were transplanted before 2008, the year in which Luminex study was routinely implemented in the transplant work-up, patients were assigned to the HS group if they had been re-transplanted and if they had lost their previous graft for rejection (two cases).

Induction was based on anti-thymocyte globulins, monoclonal anti-CD25 antibodies, or omitted according to the immunological risk. Maintenance was based on tacrolimus, mycophenolate, and steroids. Desensitization was based on anti-CD20 monoclonal antibodies (Rituximab, Mabthera, Roche, Basel, Switzerlad, 1 or 2 doses of 400 mg) and on plasma exchanges (total number according to DSA titer or repeated XM measurements), followed by intravenous immunoglobulins every two exchanges (Plangamma, 200 mg/Kg for session, Institute Grifols, Barcelona, Spain).

The three time-points examined in the retrospective study were as follows: (i) transplantation day; (ii) first biopsy (either for indication or for-protocol); and (iii) one-year after transplantation. In our institution, kidney biopsies are performed per-protocol at 3 and 12-months after kidney transplantation. Regarding the second time-point, we examined the sample of the first per-indication biopsy in those cases in which rejection developed before the 3-month per-protocol biopsy. In all the other cases, the 3-month per-protocol biopsy sample was studied. This choice was made in order to avoid the potential effects of B cell depleting treatment on BEVs content of the second time-point. Pathological examination and diagnosis of ABMR were updated according to Banff 2017 criteria (17). Treatment of ABMR was based on anti-CD20 monoclonal antibodies (Rituximab, Mabthera, Roche, Basel, Switzerlad, 1 dose of 400 mg at the beginning and 1 dose at the end of the cycle of 400 mg) and 6 plasma exchanges, followed by intravenous immunoglobulins every two exchanges (Plangamma, 200 mg/kg for session, Institute Grifols, Barcelona, Spain).

Regarding the prospective cohort, all patients were transplanted from 2018 to 2019, with the same desensitization, induction, and maintenance protocol employed as in the retrospective cohort.

Extracellular vesicles were studied according to the MISEV guidelines of 2018 (18). A checklist with all the items can be retrieved in Supplementary Material.

### Isolation of EVs by Sepharose-Based Size-Exclusion Chromatography

Stored serum samples at −80°C were thawed in ice. After two centrifugation steps at 2000 g, to eliminate cellular debris,1 ml of the serum was loaded on a Sepharose column, as previously described (19, 20). Concisely 10 ml syringes (Becton Dickinson; San Jose, United States) were stacked with 10 ml of Sepharose CL-2B (GE Healthcare; Uppsala, Sweden). Sepharose was previously washed with 0.32% sodium citrate PBS 0.22 μm-filtered. The tip of the syringe was stuffed with nylon stockings (40 denier). Immediately after sample load, it was followed by elution with 0.32% sodium citrate PBS 0.22μm-filtered and 18 fractions of 0.5 ml volume were sequentially collected. These fractions were analyzed through a spectrophotometer at 280 nm absorbance to estimate protein contents (NanodropTM, ThermoFisher; Waltham, MA, USA).

### Characterization of EVs by Flow Cytometry

The following step was the coupling of fraction 6–13 (50 μl for sample) to 5 μl of latex beads (4 μm in diameter) for 15 min and then with 1 ml of BCB buffer (PBS 0.1% BSA). After 1 night rotation at room temperature, fractions were incubated in a 96-well plate with two primary antibodies against the EV-specific tetraspanins biomarkers CD9 and CD81 (final dilution 1:10) (20). After careful washing and decanting, wells were charged with 200 μl of PBS 0.22μm-filtered before flow-cytometry study with the FACS-Fortessa cytometer (BD Biosciences; San Jose, CA, United States) was performed. For every well, 10.000 beads for sample were examined and MFI was used to calculate the content of EVs in the different fractions (Supplementary Figure 1). Those fractions that proved to be enriched in EVs by high expression of CD9 and CD81 were later pooled and analyzed for markers of B cells (CD19 and HLA-II) (21). The MFI value for CD19 and HLA-II was normalized to the CD9 biomarker of exosomes analyzed in the same pool. All antibodies for exosome characterization are indicated in Supplementary Table 1.

### Nanoparticle Tracking Analysis (NTA)

Size distribution and concentration of EVs were measured using the NanoSight LM10 instrument (Malvern, United Kingdom), equipped with a 638 nm laser and CCD camera (model F-033). Data were analyzed with the Nanosight NTA Software version 3.1 (build 3.1.46). Representative samples were evaluated in sterile-filtered PBS 1X (*n* = 3). Readings were taken in single capture or triplicates for 60 s at 30 frames per second, and through manual monitoring of temperature (Supplementary Figure 2).

### Complement-Dependent Cytotoxicity and Flow Cytometry Cross-Match

Complement-Dependent Cytotoxicity (CDC) crossmatch was performed with peripheral blood mononuclear cells (PBMCs) of the donor, according to the NIH Technique. To rule out the presence of autoantibodies, all crossmatch tests were carried out with sera, with and without dithiothreitol treatment, in order to discard IgM. The T and B cell flow cytometry crossmatch was performed using freshly obtained PBMCs from the donor as well. The T and B cells were identified using mouse anti-human CD19 and CD3 antibodies. Goat antihuman F(ab')2 IgG(γ) was used to identify anti-HLA IgG attached to the cells (Clones indicated in Supplementary Table 2). The shift of the median channel fluorescence (SMCF) between the test sera and the negative control sera was used to assign positivity or negativity. The cut-off values were assigned according to the median + 3 standard deviations of 20 non transfused male Single Antigen test negative sera.

### Assessment of DSAs and Calculated PRA by Luminex Technology

Anti-HLA antibodies were assessed before transplantation and during the per-protocol and the for-cause renal biopsy through Luminex-based technology (22). Briefly, antibodies were tested using Single Antigen Bead test (LIFECODES® Single Antigen, Immucor, Georgia, USA). An allele was considered positive if the MFI was over 1,500 and was x4 times higher than the Lowest Reactive Antigen (LRA) of the same locus. Calculated PRA (cPRA) was determined using a panel of 500 unrelated individuals from local population and was typed for A, B, C, DRB1, and DQB1 in high resolution (2 fields) by sequence-based typing.

### Flow Cytometry Analysis of PBMC and Lymph Nodes

Peripheral blood mononuclear cells (PBMCs) were isolated from patient blood samples by centrifugation on a density gradient (400 g for 30 min in Ficoll-paque PREMIUM, GE Healthcare, Madrid, Spain). Lymph nodes were mashed and passed through a 70 μm nylon cell strainer (BD Falcon) and single-cell suspensions were obtained. Cell surface markers were stained with antibodies indicated in Supplementary Table 3, according to the instructions of the manufacturer. In all samples, Aqua Live/Dead fixable dead cell kit (Thermo Fisher Scientific, Waltham, MA, USA) was used unambiguously to remove dead cells. Flow cytometry analysis was performed on a FACS Canto II (BD Biosciences, Heidelberg, Germany). Data were analyzed using FlowJo software (Tree Star, Ashland, OR, USA).

### Transmission Electron Microscopy (TEM)

A Holey Carbon support film on a 400-mesh copper grid was used. After the glow discharge, the sample was deposited onto the grid, which was mounted on a plunger (Leica EM-CPC) and blotted with Whatman No. 1 filter paper. The suspension was vitrified by rapid immersion in liquid ethane. The grid was mounted on a Gatan 626 cryo-transfer system and was inserted into the microscope. Images were obtained using a Jeol JEM 2011 cryo-electron microscope operated at 200 kV, recorded on a Gatan Ultrascan US1000 CCD camera, and were analyzed with a Digital Micrograph 1.8 (*n* = 3 per group).

### Statistical Analysis

Data are presented as number and/or percentages, mean and standard deviation, or median and interquartile range, as indicated. Statistical comparison among groups has been performed through Student's *t-*test, Mann-Whitney, or one-way ANOVA with LSD *post-hoc* analysis, as appropriate depending on data distribution and number of groups. Correlation between continuous variables has been explored with Pearson's analysis. Time-dependent association with rejection of the biomarkers in the retrospective analysis has been analyzed with mixed model linear rejection. All statistical tests have been conducted with a 95% confidence interval, and a *p* < 0.05 has been considered significant and has been highlighted with an asterisk in the Figures. To carry out all the above-mentioned analysis, software SPSS v.20 (SPSS Inc., Chicago, IL, USA) and GraphPad v.5 (GraphPad Software, La Jolla, CA, USA) have been used. The local Ethical Committee approved the study.

## Results

### Baseline Characteristics of the Studied Population

B cell-derived extracellular vesicles (BEVs) were extracted from stored serum samples of 30 LDKT recipients in the following time-points: transplantation day, first biopsy (either per-protocol or per-cause), and 12-month protocol biopsy. This population included the study group of 11 desensitized patients for a positive cross-match (DS group), while control groups were represented by 10 hypersensitized patients (defined as baseline cPRA I+II > 85%) not submitted to desensitization (HS group), and nine low-risk patients (defined as baseline cPRA I+II <10%, CT group). In the DS group, before starting the desensitization protocol, a stored serum sample was also available and studied in seven of the 11 patients. One patient of the DS group died of metastatic colon cancer before completing the 12-month follow-up. Baseline characteristics revealed no differences among groups in terms of age, sex, diabetes, hypertension, and donor characteristics. Calculated PRA (cPRA) I+II at baseline was higher in the HS and DS group as expected. Causes for desensitization in the DS group were a positive flow-cytometry crossmatch in 8 patients and DSA in 3 patients. Of the 11 patients, 10 had a detectable DSA by Luminex before transplantation (mean MFI 6,130 ± 3,405) and the only patient without a DSA had a positive flow-cytometry crossmatch for both T and B cells. Induction was most frequently based on lymphocyte-depleting agents in the HS and DS group, while baseline immunosuppression was based on tacrolimus, mycophenolate, and prednisone in all groups (Table 1).

**Table 1:** Baseline characteristics (above) and B cell-derived extracellular vesicles (BEVs) assessment (below) in the three study groups.

	**Control (CT) ** ***n =* 9**	**Hypersensitized (HS) *n =* 10**	**Desensitized (DS) *n =* 11**
Age (years)	41.9 ± 9.94	43.2 ± 6.86	45.4 ± 15.64
Sex (%males)	3/9 (33.3%)	4/10 (40.0%)	5/11 (45.5%)
Dialysis vintage (months)	20.8 ± 22.3	65.8 ± 52.3	49.3 ± 78.1
Hypertension (% yes)	8/9 (88.8%)	8/10 (80.0%)	9/11 (81.8%)
Diabetes mellitus (% yes)	2/9 (22.2%)	1/10 (10.0%)	3/11 (27.3%)
Previously transplanted (% yes)	2/9 (22.2%)	5/10 (50.0%)	6/11 (54.5%)
Donor age (years)	52.0 ± 14.2	55.3 ± 13.3	54.4 ± 10.3
Donor sex (%males)	5/9 (55.5%)	4/10 (40.0%)	4/11 (36.4%)
HLA A-B-DR incompatibilities	3.11 ± 1.90	2.20 ± 2.46	3.91 ± 1.57
cPRA I+II at baseline (%)	0	95.6 ± 3.8	78.4 ± 21.4
Induction (%)			
None	1/9 (11.1%)	/	/
Anti-CD25 antibodies	5/9 (55.5%)	1/10 (10.0%)	/
Lymphocyte-depleting agents	3/9 (33.3%)	9/10(90.0%)	11/11 (100.0%)
Creatinine +3 months (mg/dl)	1.22 ± 0.48	1.59 ± 0.49	1.55 ± 0.60
Creatinine +12 months (mg/dl)	1.23 ± 0.53	1.65 ± 0.58	1.69 ± 0.58
**Desensitization parameters**			
DSA (MFI)	/	/	6,130 ± 3,405
Rituximab (mg)	/	/	618.2 ± 208.9
Plasma exchanges (*n*)	/	/	5 [2–7]

*HLA, Human Leukocyte Antigen; cPRA, calculated Panel-Reactive Antibodies*.

### Circulating BEVs Can Be Detected During the First Year After Kidney Transplantation

**Figure 1:**
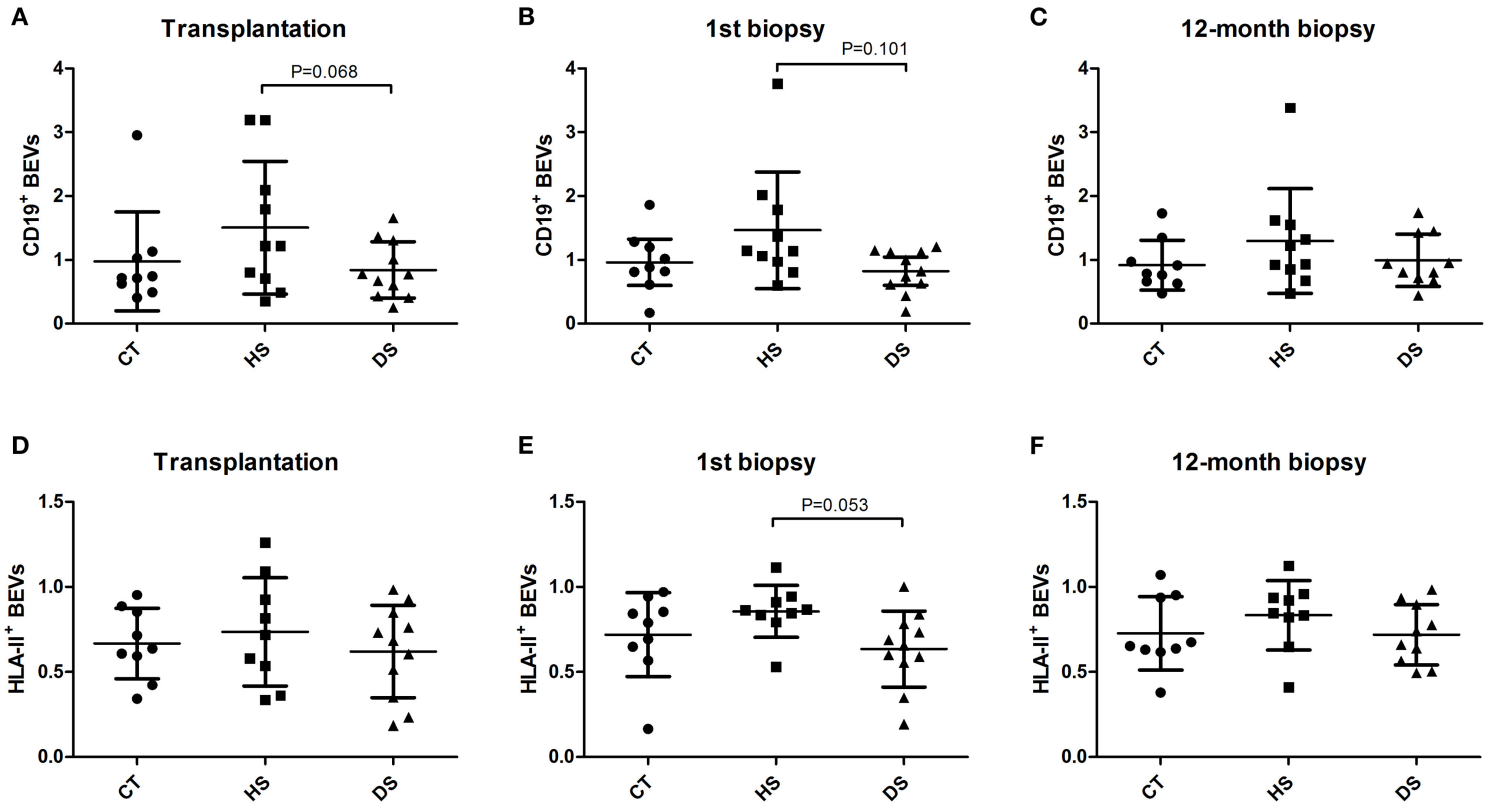
B cell-derived Extracellular Vesicles (BEVs) evolution along the first year after kidney transplantation at the time of Transplantation **(A,D)**, first biopsy **(B,E)** and 12-month protocol biopsy **(C,F)**. BEVs (CD19^+^ and HLA-II^+^ EVs) were analyzed in the study groups in the three time-points. transplantation day, first biopsy, and 12-month protocol biopsy (ANOVA and LSD *Post-hoc* analysis).

Isolation and characterization of microparticles were consistent with EV surface biomarkers (CD9 and CD81) and with the diameter according to flow-cytometry (Supplementary Figure 1), NTA analysis (Supplementary Figure 2) and electronic Microscopy (Supplementary Figure 3). BEVs were defined as either CD19+ or HLA-II+ EVs and were detected all along the first year after the kidney transplantation in the three groups (CT, HS, and DS) (Figure 1), without significant difference at ANOVA analysis (Table 2). However, when comparing the DS group with the similar risk controls (HS group), there was a tendency toward lower CD19+ EVs at transplantation (*p* = 0.068, Figure 1A), at first biopsy (*p* = 0.101, Figure 1B), and lower HLA-II+ EVs at first biopsy (*p* = 0.053, Figure 1E).

**Table 2:** BEVs assessment (below) in the three study groups at transplantation, first renal biopsy, and at 12-month protocol biopsy.

	**Control (CT) *n =* 9**	**Hypersensitized (HS) *n =* 10**	**Desensitized (DS) *n =* 11**	***P*-value (ANOVA)**
**Transplantation (BEVs)**				
CD19^+^ EVs	0.97 ± 0.77	1.50 ± 1.04	0.84 ± 0.44	0.148
HLA-II^+^ EVs	0.66 ± 0.20	0.73 ± 0.31	0.62 ± 0.27	0.639
**First renal biopsy (BEVs)**				
CD19^+^ EVs	0.96 ± 0.47	1.46 ± 0.91	0.88 ± 0.40	0.102
HLA-II^+^ EVs	0.71 ± 0.24	0.85 ± 0.15	0.64 ± 0.22	0.097
**12-month renal biopsy (BEVs)**				
CD19^+^ EVs	0.91 ± 0.39	1.29 ± 0.50	0.99 ± 0.40	0.337
HLA-II^+^ EVs	0.72 ± 0.21	0.83 ± 0.20	0.71 ± 0.17	0.403

### BEVs Drop After Desensitization and Relapse During ABMR

Antibody-mediated rejection (ABMR) during the first year after kidney transplantation occurred in seven of the 11 patients of the DS group. Of the seven rejections in the DS group, four were discovered at 3-month protocol biopsy, and three were discovered before in a per-cause renal biopsy. Within this group, DSAs were present in two of seven patients with ABMR (MFI of 3,654 and 4,077, respectively) and in two of four patients without ABMR (MFI of 9,066 and 6,613, respectively).

**Figure 2:**
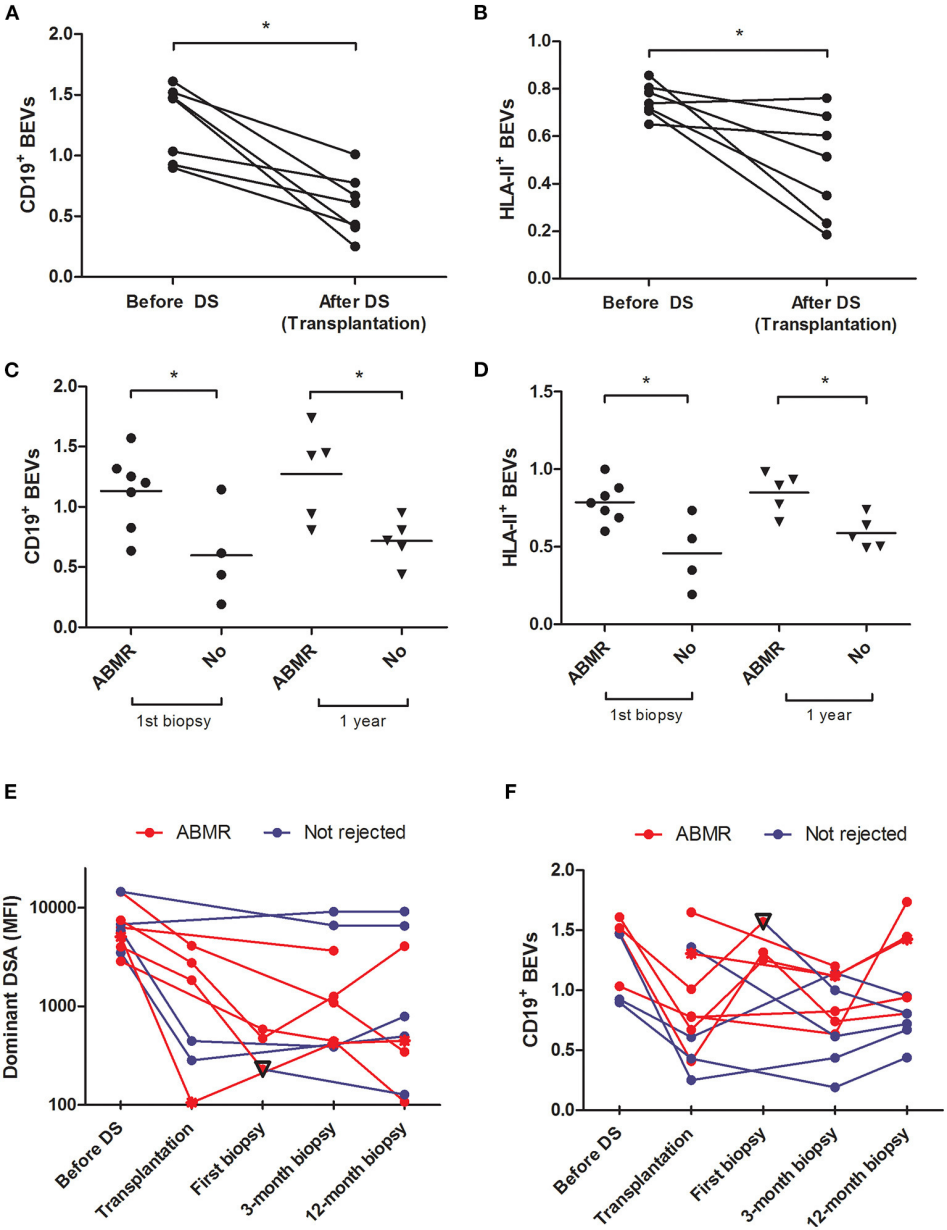
Analysis of BEVs in patients undergoing desensitization and their relationship with antibody-mediated rejection (ABMR)–BEVs expressed as CD19^+^ and HLA-II^+^ exosomes before and after desensitization in the DS group [**(A,B)** paired samples *t*-test] and according to whether or not patients developed ABMR at first biopsy or at 12 months after kidney transplantation [**(C,D)** Student's *t*-test, Table 3]. Evolution of patients according to whether they have rejected (red line) or not (blue line) during the first year is highlighted for MFI of the DSA **(E)** and CD19^+^BEVs **(F)**. Among patients with ABMR, the only patient who did not progress to chronic ABMR and had a normal kidney at following biopsies is highlighted with a reverse triangle.

We observed a significant drop in both CD19+ and HLA-II+ EVs after desensitization (CD19+ EVs 1.27 ± 0.30 before and 0.59 ± 0.25 after desensitization, *p* = 0.003, and HLA-II+ EVs 0.75 ± 0.06 before and 0.47 ± 0.22 after desensitization, *p* = 0.024) (Figures 2A,B). Within the DS group, patients with active ABMR had higher CD19+ and HLA-II+ EVs both at first biopsy (*n* = 7) and at 12 months (*n* = 5) (Table 3 and Figures 2C,D). All the cases of ABMR were treated except the patient with neoplasia. Only one patient experienced full recovery after treatment, while the other patients progressed to chronic ABMR at 12-month protocol biopsy. Evolution of CD19+ EVs after transplantation proved to be significantly associated with rejection (*p* = 0.002), while DSAs were not (*p* = 0.186), according to mixed-model linear rejection analysis (Figures 2E,F).

**Table 3:** BEVs evolution in patients submitted to desensitization according to the clinical outcome.

		***n***	**CD19^**+**^ EVs**	***P*-value**	**HLA-II^**+**^ EVs**	***P*-value**
First biopsy						
	No rejection	4	0.59 ± 0.40	0.036	0.45 ± 0.23	0.014
	ABMR	7	1.13 ± 0.31		0.78 ± 0.13	
12-month biopsy						
	No rejection	5	0.71 ± 0.18	0.021	0.58 ± 0.10	0.008
	ABMR	5^*^	1.27 ± 0.38		0.84 ± 0.13	

* *1 missing value (patient death).*

*ABMR, antibody-mediated rejection*.

### Survival of CD19+CD20- B Cells in Lymph Nodes After Desensitization

We developed a prospective validation study to assess the B cell subpopulations from PBMC and the external iliac lymph node recovered upon surgery, along with the circulating BEVs. In this cohort, we studied 11 patients of which six were submitted to the same desensitization protocol as above, while five low-risk recipients represented the control group. Baseline characteristics of this prospective population are described in Table 4, without any significant difference between the two groups.

**Table 4:** Baseline characteristics of the prospective validation study cohort.

	**Desensitized *n =* 6**	**Control** ***n =* 5**	***P*-value**
Age (years)	51.2 ± 12.7	45.2 ± 20.0	0.562
Sex (% males)	6 (100.0%)	4 (80.0%)	0.455
Dialysis vintage (months)	0 [0–48]	8 [3–17]	0.840
Hypertension (% yes)	5 (83.3%)	5 (100.0%)	1
Diabetes mellitus (% yes)	1 (16.7%)	0 (0.0%)	1
Previously transplanted (% yes)	1 (16.7%)	1 (20.0%)	1
Donor age (years)	54.7 ± 4.96	56.6 ± 4.21	0.510
Donor sex (% males)	0 (0.0%)	0 (0.0%)	1
HLA A-B-DR incompatibilities	4.00 ± 2.44	3.00 ± 2.23	0.501
cPRA I+II at baseline (%)	0 [0–100]	0 [0–46]	0.714
Creatinine +3 months (mg/dl)	1.49 ± 0.48	1.52 ± 0.37	0.926
Creatinine +6 months (mg/dl)	1.44 ± 0.28	1.77 ± 0.59	0.420
**Desensitization parameters**			
Isoaglutinins IgG	4 [2–32]		
Isoaglutinins IgM	4 [2–64]		
Rituximab (mg)	1,000.0 ± 903.3		
Plasma exchanges (*n*)	4 [1–7]		

*The desensitized group also included ABO-incompatible recipients, so data about pre-transplant isoaglutinins IgG and IgM had been added.*

*HLA, Human Leukocyte Antigen; cPRA, calculated Panel-Reactive Antibodies*.

**Figure 3:**
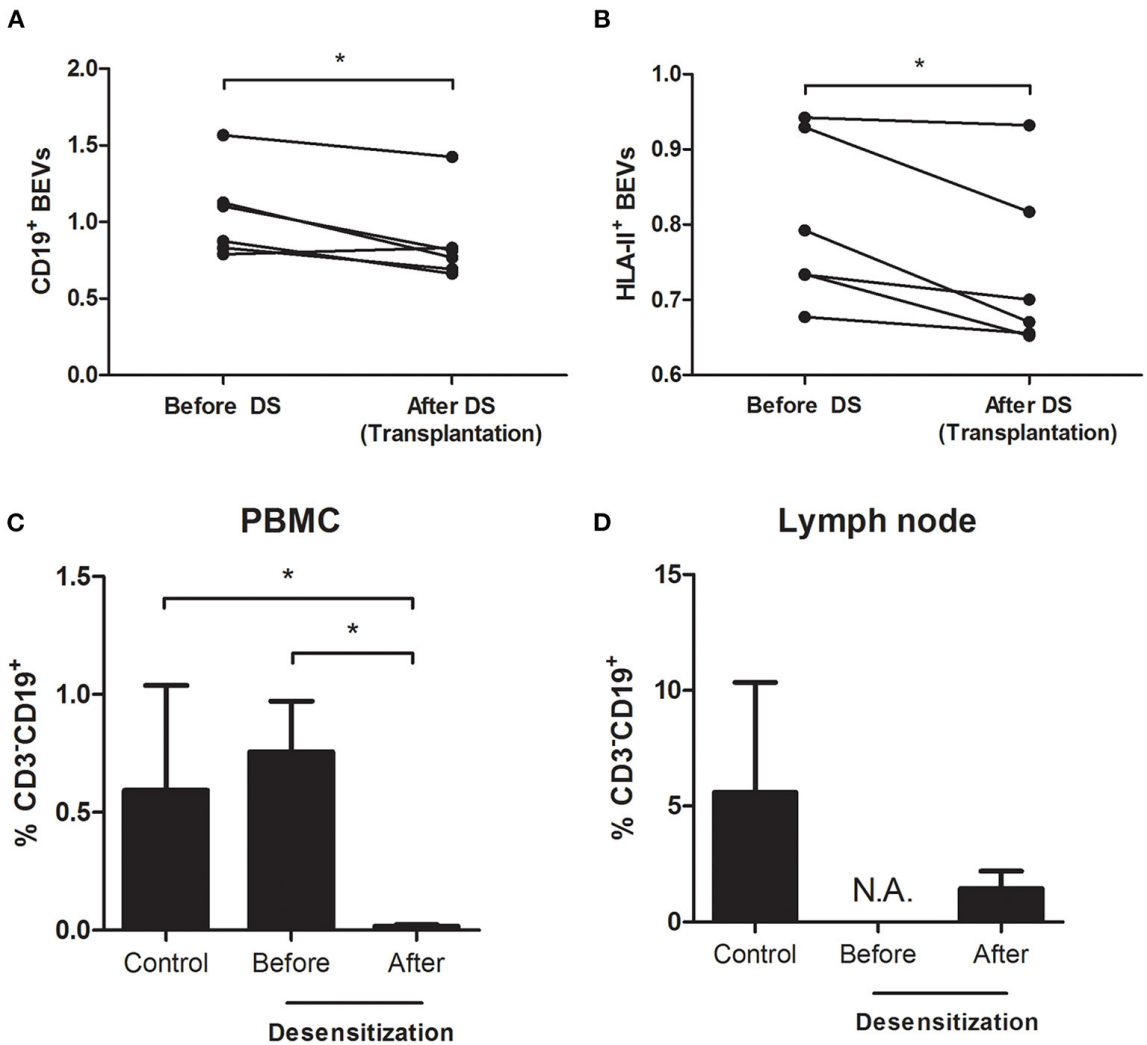
Analysis of BEVs and B cells from peripheral blood mononuclear cells (PBMCs) and lymph node in the prospective validation cohort–CD19^+^ and HLA-II^+^ BEVs before and after desensitization [**(A,B)** paired-samples t-test]. At baseline, there is no difference in percentage of circulating B cells (CD3^−^CD19^+^) between the two groups of patients. Upon desensitization, there is a depletion in circulating CD3^−^CD19^+^ cells [**(C)** Student's t-test] but a relevant proportion of them persisted alive in the lymph node **(D)**.

**Figure 4:**
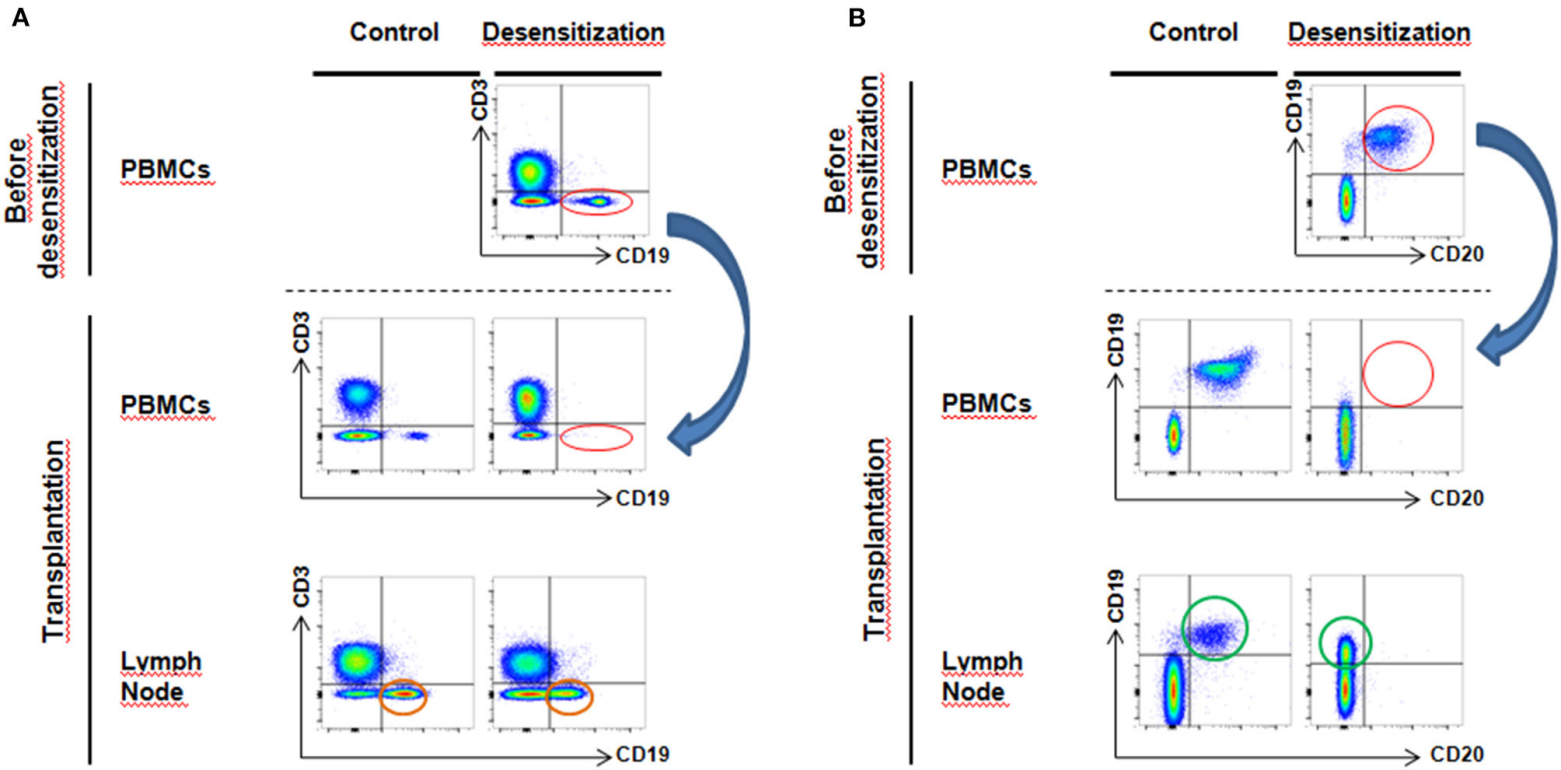
Flow cytometry analysis of circulating and lymph node B cells–**(A)** After desensitization, CD3^−^CD19^+^ B cells disappear from blood circulation (red circle, blue arrow). However, in lymph nodes at the moment of transplantation, we still observe a substantial proportion of surviving CD3^−^CD19^+^ B cells (orange circle). **(B)** Desensitization eliminates all CD19^+^CD20^+^ B cells from peripheral circulation (red circle, blue arrow). However, at transplantation, we observe that the CD19^+^CD20^+^ population lose CD20 expression compared to the control group (green circle, CD19^+^CD20^−^).

There was a total depletion of CD3-CD20+ B cells from both periphery and lymph node after desensitization. The CD3-CD19+ B cells were almost depleted after desensitization in blood (Figure 3C, *p* = 0.024), but they were still present in the lymph node, without statistical difference compared to the control group at the time of transplantation (Figure 3D, *p* = 0.762, and representative sample in Figure 4A). On the other side, CD19+CD20+ B cells were also undetectable in lymph nodes in the DS group, while a substantial presence of CD19+CD20- B cells still persisted (Figure 4B).

### Circulating BEVs Correlate With Residual CD3-CD19+ B Cells in Lymph Nodes That Display a Switched-Memory Phenotype

We confirmed the significant drop in BEVs after desensitization, being CD19+ EVs 1.04 ± 0.29 before and 0.80 ± 0.11 after (*p* = 0.024) and HLA-II+ EVs 0.86 ± 0.28 before and 0.73 ± 0.11 after desensitization (*p* = 0.024), respectively (Figures 3A,B). There was a significant correlation between CD19+ EVs and the percentage of CD3-CD19+ B cells in the lymph node (*r* = 0.839, *p* = 0.037) at the moment of transplantation, while there was no correlation between circulating CD19+ PBMCs and CD3-CD19+ B cells in the lymph node (*r* = −0.197, *p* = 0.708).

**Figure 5:**
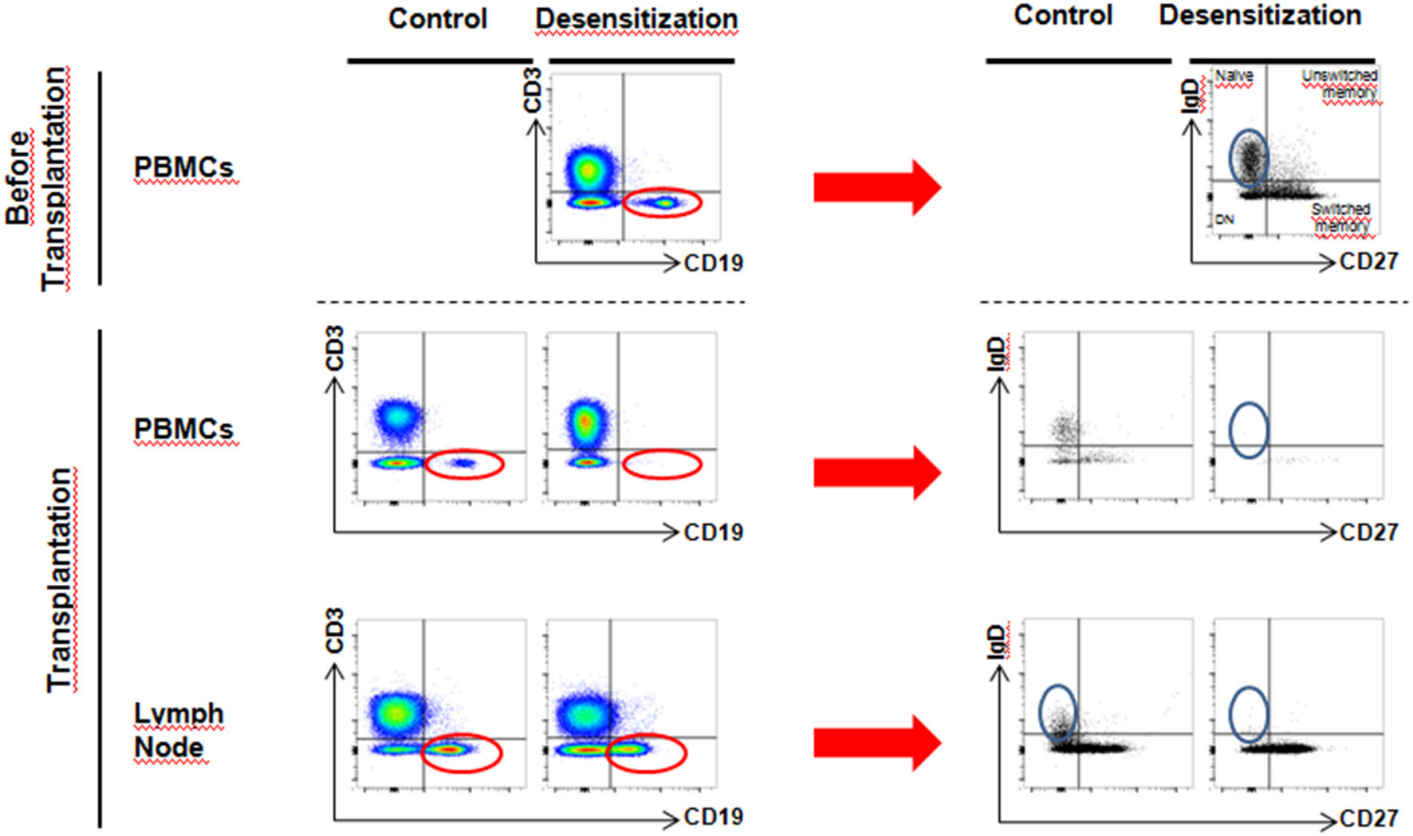
More detailed phenotype analysis of circulating and lymph node B cells–By selection of CD3^−^CD19^+^ cells (red circle) and further analysis of phenotype, we observed a loss of naive B cells (CD27^−^IgD^+^) upon desensitization in the lymph node (blue circle), while CD27^+^IgD^−^ cell population still persisted (switched memory phenotype).

More precise phenotype of B cells revealed that after desensitization, there was a loss of naïve B cells (CD27-IgD+) in lymph nodes, while a substantial proportion of switched-memory B cells (CD27+IgD-) was still alive (Figure 5).

## Discussion

Desensitization before LDKT represents a reasonable option for highly sensitized patients in terms of graft and patient survival (4). However, ABMR incidence can be as high as 30–50% (5–7). This suggests that the biomarkers currently used to check proper desensitization (DSAs, circulating B cells, flow-cytometry crossmatch) only partially reflect the complex biology of humoral alloimmunity. In order to check B cell proliferation in secondary lymphoid organs and bone marrow after desensitization, we propose the use of B cells-derived EVs.

B cell-derived extracellular vesicles (BEVs) are generated by B cells upon differentiation and proliferation stimuli (13) and could be a major source of EVs *in vivo* (11). In large B cell lymphomas, exosome-derived miRNAs are associated with chemotherapy resistance (17). The value of circulating EVs as diagnostic tool has been highlighted in oncology (14) and in kidney transplantation (15, 16). A recent report also found out that urinary CD3+ EVs are associated with cellular rejection (23). To date, there are no studies that specifically assessed BEVs in kidney transplant recipients.

In our experience, we observed the presence of circulating BEVs in normal and hypersensitized kidney transplant recipients, before and within the first year after transplantation, with a non-significant trend toward higher concentration of BEVs in the hypersensitized group (Table 2 and Figure 1).

In patients submitted to pre-transplant desensitization, we observed a significant reduction in BEVs upon treatment completion (Figures 2A,B) with a significant rebound in patients who later developed ABMR (Table 3 and Figures 2C,D). In this prospective study, we observed a complete depletion of CD3-CD20+ both in periphery and in the lymph node. B cells were also assessed as CD3-CD19+ and we also observed that these were almost completely cleared from circulation (Figure 4A). In lymph nodes, however, we observed a switch from CD19+CD20+ to CD19+CD20- phenotype in desensitized patients (Figure 4B), with a switched-memory phenotype (Figure 5) and a statistical association with circulating CD19+ EVs. Differences, albeit moderate, were statistically significant and also showed that the evolution of CD19+ EVs was associated with the development of rejection while the DSAs were not (Figures 2E,F).

Taken together, these findings confirmed that a desensitization protocol based on anti-CD20 antibodies, intravenous immunoglobulins, and plasma exchanges is associated with residual alloimmunity that cannot be detected with the currently available clinical tools. As we observed a significant increase in circulating BEVs in patients experiencing ABMR, we propose that this reflects proliferation and differentiation of alloreactive B cells resident in secondary lymphoid organs and bone marrow that would not be detectable otherwise.

However, some points need to be addressed before drawing firm conclusions, given the small sample size analyzed in this preliminary experience and the generating-hypothesis nature of this work. First, while in the DS group, there was a striking difference in BEVs before and after desensitization and between patients who rejected or not, we did not observe significant differences between the DS and the other groups (Figure 2). This may suggest that the relative changes in BEVs can be meaningful in patients treated with B cell targeted therapies, but at present, this cannot be applied to other population who receive standard-of-care induction. Second, the two biomarkers that we used to define BEVs (CD19 and HLA-II) were not specific of only a cell population. The CD19 is expressed by B cells in most of their differentiation steps and HLA-II is also expressed by other antigen-presenting cells such as monocytes-macrophages and dendritic cells. Third, as B cells lose CD19 expression along their differentiation, CD19+ EVs may not reflect the activity of plasma cells, which are ultimately responsible for DSA production. However, it seems that non-myelomatous plasma cells still express CD19, even though in a heterogeneous way between individuals (24, 25), while there is more information about EVs secretion by myelomatous plasma cells (26, 27).

In conclusion, in patients undergoing desensitization before kidney transplantation, we observed a significant decrease in circulating BEVs after treatment. These were associated with the presence of surviving B cells in the lymph nodes. Patients who developed ABMR have experienced a significant rebound in circulating BEVs, suggesting proliferation and differentiation of not-circulating alloreactive B cells. The next steps will be to analyze BEVs kinetic and function not only in desensitized patients but also in low and high-risk kidney transplant recipients. Circulating BEVs may demonstrate to reveal a part of the humoral response that cannot be assessed yet in clinical practice, i.e., alloreactive B cells resident in primary and secondary lymphoid organs.

## Data Availability Statement

The raw data supporting the conclusions of this article will be made available by the authors, without undue reservation.

## Ethics Statement

The studies involving human participants were reviewed and approved by HCB/2016/0394. The patients/participants provided their written informed consent to participate in this study.

## Author Contributions

DC was involved in study conception, laboratory experiments, data analysis, and writing of the manuscript. VT was involved in laboratory experiments and data analysis. JR, MR-B, and EB-M were involved in study conception, laboratory experiment, and data analysis. ML-R and NH-G were involved in laboratory experiments. FB, JM, and AH was involved in study conception and data analysis. PV-A and GP were involved in data analysis and writing of the manuscript. LP and MM were involved in data analysis. FO, JC, and FD were involved in data analysis and revision of the final draft of the manuscript. IR was involved in study conception, data analysis, and writing of the manuscript. All authors contributed to the article and approved the submitted version.

## Funding

This study has been funded by the research grant from Instituto de Salud Carlos III (ISCIII) under the program Acción Estratégica en Salud 2016 (PI16/00115), Redes Temáticas de Investigación Cooperativa en Salud, REDINREN (RD16/0009/0023) co-funded by ISCIII Subdirección General de Evaluación and Fondo Europeo de Desarrollo Regional (FEDER) Una manera de hacer Europa, and Secretaria d'Universitats i Recerca and CERCA Programme del Departament d'Economia i Coneixement de la Generalitat de Catalunya (2017-SGR-1331). VT received a personal grant from Fundació Catalana de Trasplantament.

## Conflict of Interest

The authors declare that the research was conducted in the absence of any commercial or financial relationships that could be construed as a potential conflict of interest.

## Publisher's Note

All claims expressed in this article are solely those of the authors and do not necessarily represent those of their affiliated organizations, or those of the publisher, the editors and the reviewers. Any product that may be evaluated in this article, or claim that may be made by its manufacturer, is not guaranteed or endorsed by the publisher.
