# Supplementary figures and images for: B Cell-Derived Extracellular Vesicles Reveal Residual B Cell Activity in Kidney Graft Recipients Undergoing Pre-Transplant Desensitization
Source: Front Med (Lausanne). 2021 Dec 16;8:781239. doi: 10.3389/fmed.2021.781239 (PMC8716735; doi:10.3389/fmed.2021.781239)

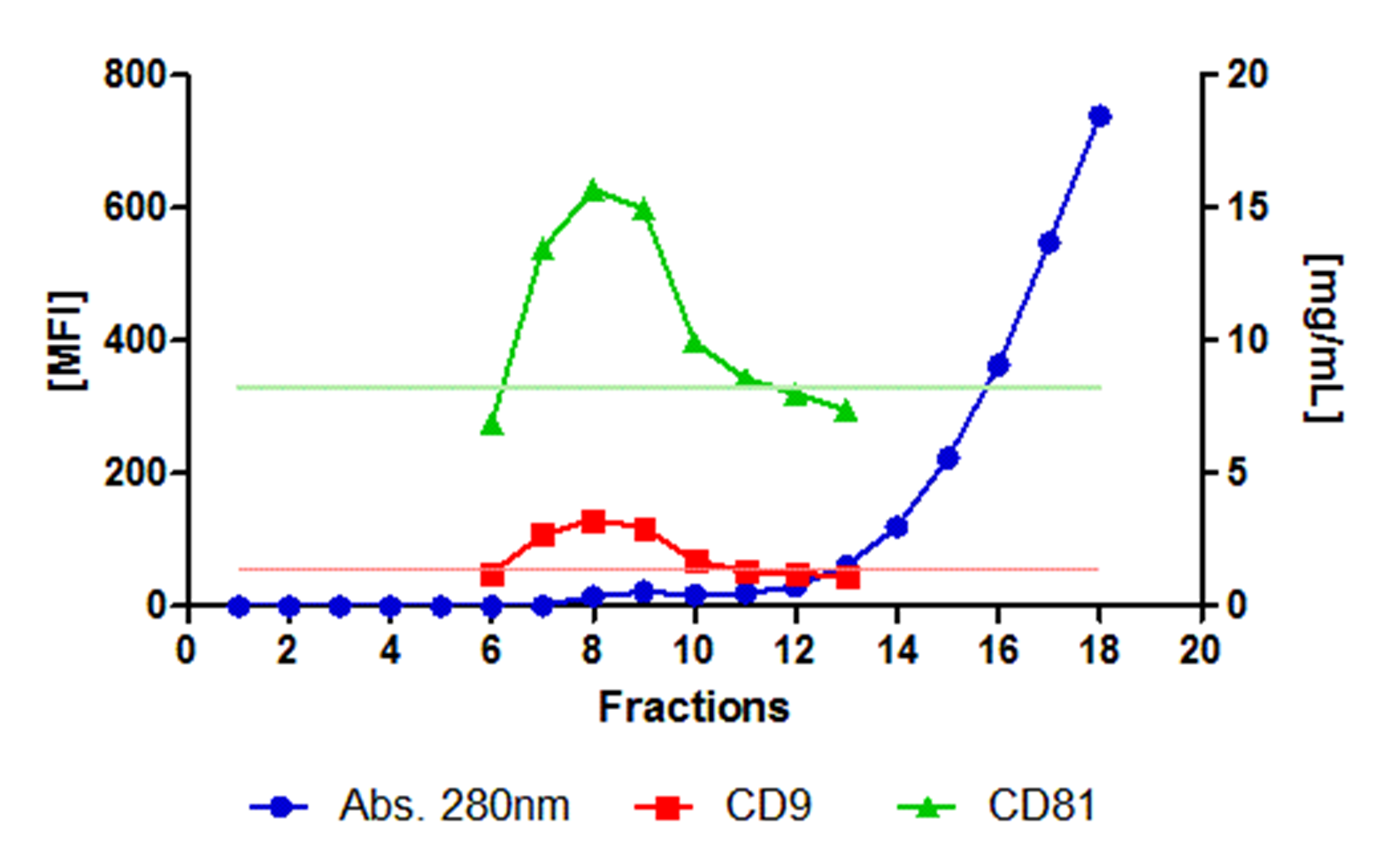

Supplement: Supplementary file 1 [file Image_1.TIF]

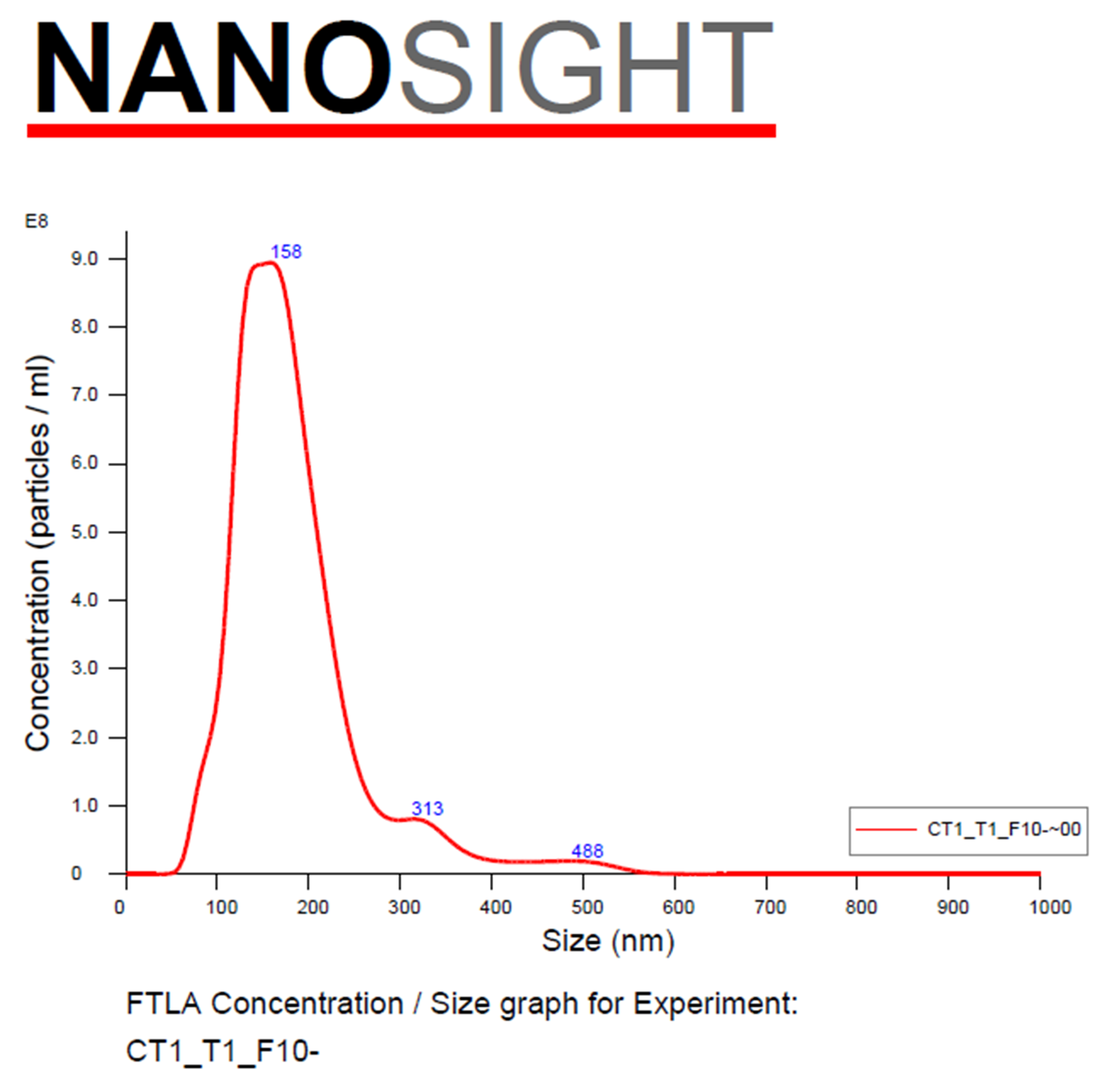

Supplement: Supplementary file 2 [file Image_2.TIF]

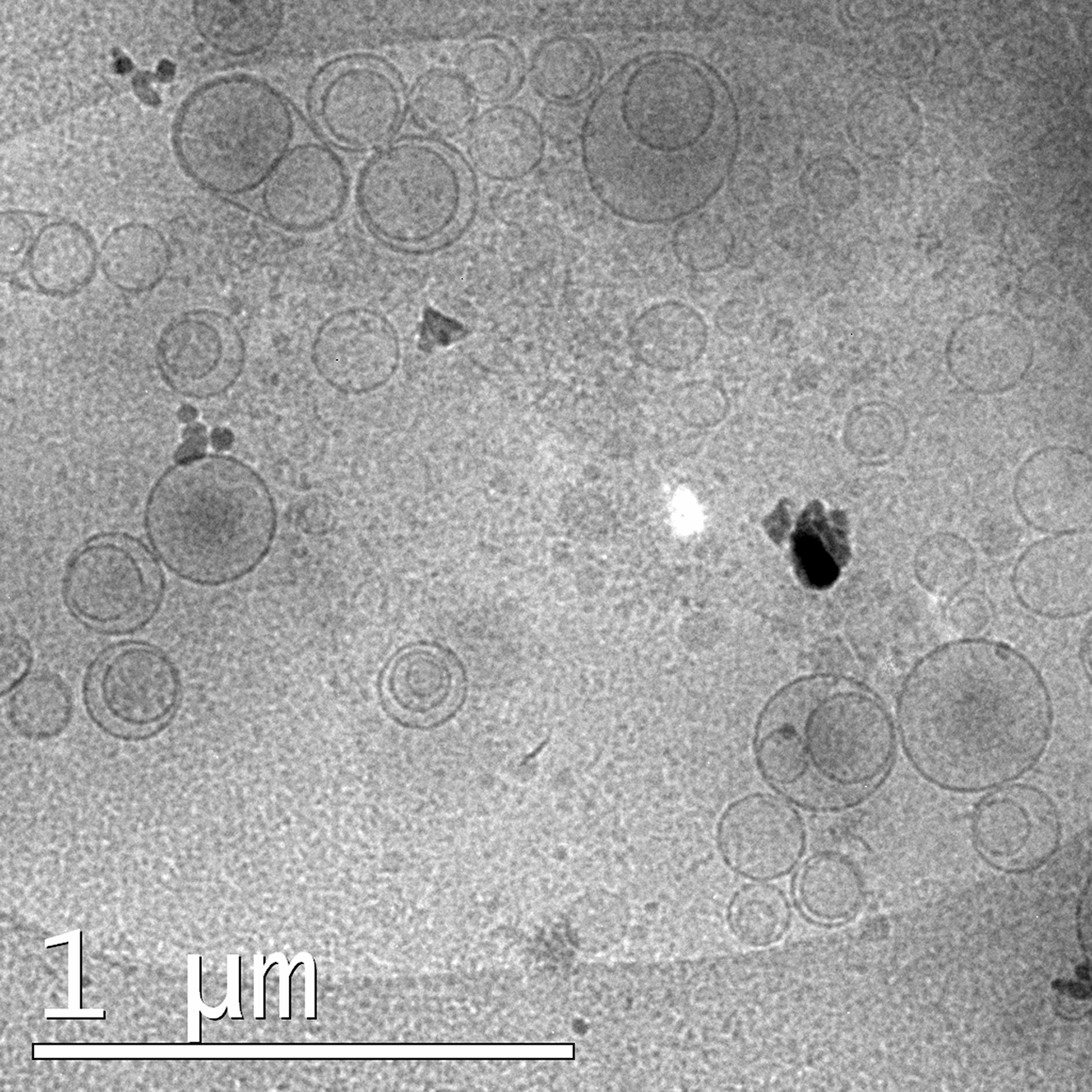

Supplement: Supplementary file 3 [file Image_3.TIF]
