# Supplementary material for: B Cell-Derived Extracellular Vesicles Reveal Residual B Cell Activity in Kidney Graft Recipients Undergoing Pre-Transplant Desensitization
Source: Front Med (Lausanne). 2021 Dec 16;8:781239. doi: 10.3389/fmed.2021.781239 (PMC8716735; doi:10.3389/fmed.2021.781239)
Supplement: Supplementary file 4 [file Data_Sheet_1.docx]

Supplementary Material

**TABLE OF CONTENTS:**

- **Table S1:** Flow cytometry antibodies for characterization of BEVs.
- **Table S2:** Antibodies employed for flow-cytometry cross-match.
- **Table S3:** Flow cytometry antibodies for lymphocyte characterization.
- **Figure S1:** Characterization of extracellular vesicles through bead-based flow cytometry
- **Figure S2:**  Characterization of extracellular vesicles through Nanoparticle Tracking Analysis (NTA)
- **Figure S3**: Electron Microscopy Analysis of isolated EVs
- **MISEV 2018 Guidelines**

**Supplementary Table S1:** Flow cytometry antibodies for characterization of BEVs.

| **Marker** | **Dye** | **Clone** | **Company** |
| --- | --- | --- | --- |
| CD9 | FITC | MEM-61 | Thermo Fisher Scientific |
| CD81 | PE | M38 | Thermo Fisher Scientific |
| CD19 | FITC | HIB19 | BD Pharmingen |
| HLA-DR,DP, DQ | FITC | REA332 | Miltenyi |
| IgG1 Isotype control | FITC | MOPC-21 | Thermo Scientific Scientific |
| IgG1 Isotype control | PE | MOPC-21 | Thermo Scientific Scientific |

**Supplementary Table S2:** Antibodies employed for flow-cytometry cross-match.

| **Marker** | **Dye** | **Clone** | **Company** |
| --- | --- | --- | --- |
| CD19 | PE | SJ25C1 | BD Biosciences |
| CD3 | PE | SK7 | BD Pharmingen |
| Anti-IgG | FITC | 31628 | Invitrogen |

**Supplementary Table S3:** Flow cytometry antibodies for lymphocyte characterization.

| Marker | Dye | Clone | Company |
| --- | --- | --- | --- |
| IgD | FITC | IA6-2 | Thermo Fisher Scientific |
| CD20 | PE | 2H7 | Thermo Fisher Scientific |
| CD27 | PerCP-eFluor 710 | LG.7F9 | Thermo Fisher Scientific |
| CD19 | APC | SJ25C1 | Thermo Fisher Scientific |
| CD3 | eFluor 450 | OKT3 | Thermo Fisher Scientific |
| CD24 | PE-Cy7 | eBioSN3 | Thermo Fisher Scientific |
| CD45RO | APC | UCHL1 | Thermo Fisher Scientific |
| CD19 | APC-eFluor780 | HIB19 | Thermo Fisher Scientific |
| CD16 | FITC | B73.1 | BD Bioscience |
| TCRαβ | PE | IP26 | Thermo Fisher Scientific |
| CD3 | PerCP-eFluor 710 | OKT3 | Thermo Fisher Scientific |
| CD8α | PE-Cy7 | SK1 | Thermo Fisher Scientific |
| CD14 | APC | MφP9 | BD Bioscience |
| CD4 | APC-eFluor780 | OKT4 | Thermo Fisher Scientific |

**FIGURE LEGENDS**

**Figure S1** – Characterization of extracellular vesicles through bead-based flow cytometry - Content in proteins (mg/ml, 280-nm absorbance) and EVs (CD9^+^ and CD81^+^) calculated as MFI expression through beads-based flow cytometry in 18 consecutive fractions obtained by eluting a representative sample by means of size-exclusion chromatography based on Sepharose. Control isotype is expressed as a pale line of the same color as that of CD9 and CD81.

**Figure S2**  - Characterization of extracellular vesicles through Nanoparticle Tracking Analysis (NTA) – Representative sample of EVs purified by size-exclusion chromatography from human serum samples analyzed by NTA. The mean size of EVs was 183 ± 73 nm.

**Figure S3**: Electron Microscopy Analysis of isolated EVs - Transmission Electron Microscopy analysis of the EVs extracted from human serum using SEC isolation method.

**1-Nomenclature**

***Mandatory***

+++ Generic term extracellular vesicle (**EV**): **With demonstration** of **extracellular** (no intact cells) and **vesicular** nature per these characterization (Section 4) and function (Section 5) guidelines **OR – COMPLIED -**

+++ Generic term, e.g., extracellular particle (**EP**): no intact cells but MISEV guidelines not satisfied

***Encouraged (choose one)***

+ Generic term extracellular vesicle (**EV**) + **specification** (size, density, other) **– COMPLIED -**

+ Specific term for subcellular origin: e.g., ectosome, microparticle, microvesicle (from plasma membrane), exosome (from endosomes), **with demonstration** of the subcellular origin

+ Other specific term: **with definition of specific criteria**

**2-Collection and pre-processing**

***Biofluids or Tissues (Sections 2-b and -c)***

 ++ Donor status if available (age, sex, food/water intake, collection time, disease, medication, other) **– COMPLIED -**

 +++ Volume of biofluid or volume/mass of tissue sample collected per donor **– COMPLIED -**

 ++ Total volume/mass used for EV isolation (if pooled from several donors) **– NOT APPLICABLE -**

 +++ All known collection conditions, including additives, at time of collection **– COMPLIED -**

 +++ Pre-treatment to separate major fluid-specific contaminants before EV isolation **– COMPLIED -**

 +++ Temperature and time of biofluid/tissue handling before and during pre-treatment **– COMPLIED -**

 ++ For cultured tissue explants: volume, nature of medium and time of culture before collecting conditioned medium **– NOT APPLICABLE -**

 ++ For direct tissue EV extraction: treatment of tissue to release vesicles without disrupting cells **– NOT APPLICABLE -**

***Storage and recovery (Section 2-d)***

 +++ Storage and recovery (e.g., thawing) of CCM, biofluid, or tissue before EV isolation (storage temperature, vessel, time; method of thawing or other sample preparation) **– COMPLIED -**

 +++ Storage and recovery of EVs after isolation (temperature, vessel, time, additive(s)…) **– COMPLIED -**

**3-EV separation and concentration**

***Experimental details of the method -* – COMPLIED for SEPHAROSE-BASED SIZE-EXCLUSION CROMATOGRAPHY-**

 ++ Centrifugation: reference number of tube(s), rotor(s), adjusted k factor(s) of each centrifugation step (= time+ speed+ rotor, volume/density of centrifugation conditions), temperature, brake settings

 ++ Density gradient: nature of matrix, method of generating gradient, reference (and size) of tubes, bottom-up (sample at bottom, high density) or top-bottom (sample on top, low density), centrifugation speed and time (with brake specified), method and volume of fraction recovery

 ++ Chromatography: matrix (nature, pore size,…), loaded sample volume, fraction volume, number

 ++ Precipitation: reference of polymer, ratio vol/vol or weight/vol polymer/fluid, time/temperature of incubation, time/speed/temperature of centrifugation

 ++ Filtration: reference of filter type (=nature of membrane, pore size…), time and speed of centrifugation, volume before/after (in case of concentration)

 ++ Antibody-based : reference of antibodies, mass Ab/amount of EVs, nature of Ab carrier (bead, surface) and amount of Ab/carrier surface

 ++ Other…: all necessary details to allow replication

 ++ Additional step(s) to concentrate, if any

 ++ Additional step(s) to wash matrix and/or sample, if any

***Specify category of the chosen EV separation/concentration method (Table 1):***

 + High recovery, low specificity = mixed EVs and non-EV components **OR**

**+ Intermediate recovery, intermediate specificity = mixed EVs with limited non-EV components OR – COMPLIED -**

 + Low recovery, high specificity = subtype(s) of EVs with as little non-EV as possible **OR**

 + High recovery, high specificity = subtype(s) of EVs with as little non-EV as possible

**4-EV characterization**

***Quantification (Table 2a, Section 4-a)***

 +++ Volume of fluid, and/or cell number, and/or tissue mass used to isolate EVs **– COMPLIED -**

 +++ Global quantification by at least 2 methods: protein amount, particle number, lipid amount, expressed per volume of initial fluid or number of producing cells/mass of tissue - **– NOT COMPLIED -**

 +++ Ratio of the 2 quantification figures - **NOT COMPLIED -**

***Global characterization (Section 4-b, Table 3)***

 +++ Transmembrane or GPI-anchored protein localized in cells at plasma membrane or endosomes **– COMPLIED -**

 +++ Cytosolic protein with membrane-binding or -association capacity **– NOT COMPLIED -**

 +++ Assessment of presence/absence of expected contaminants **– COMPLIED -**

 (At least one each of the three categories above)

 ++ Presence of proteins associated with compartments other than plasma membrane or endosomes

 ++ Presence of soluble secreted proteins and their likely transmembrane ligands

 + Topology of the relevant functional components (Section 4-d)

***Single EV characterization (Section 4-c)***

 +++ Images of single EVs **by wide-field and close-up**: e.g. electron microscopy, scanning probe microscopy, super-resolution fluorescence microscopy – **COMPLIED -**

 +++ Non-image-based method analysing large numbers of single EVs: NTA, TRPS, FCS, high-resolution flow cytometry, multi-angle light-scattering, Raman spectroscopy, etc. **– COMPLIED (NTA) -**

**5-Functional studies – NOT APPLICABLE -**

 +++ Dose-response assessment

 +++ Negative control = nonconditioned medium, biofluid/tissue from control donors, as applicable

 +++ Quantitative comparison of functional activity of total fluid, vs EV-depleted fluid, vs EVs (after high recovery/low specificity separation)

 +++ Quantitative comparison of functional activity of EVs vs other EPs/fractions after low recovery/high specificity separation

 + Quantitative comparison of activity of EV subtypes (if subtype-specific function claimed)

 + Extent of functional activity in the absence of contact between EV donor and EV recipient

**6-Reporting**

 + Submission of methodologic details to EV-TRACK (evtrack.org) with EV-TRACK number provided (strongly encouraged) **– NOT COMPLIED -**

 +++ Submission of data (proteomic, sequencing, other) to relevant public, curated databases or open-access repositories **– NOT APPLICABLE-**

 + Data submission to EV-specific databases (e.g., EVpedia, Vesiclepedia, exRNA atlas) **– NOT COMPLIED -**

 ++ Temper EV-specific claims when MISEV requirements cannot be entirely satisfied (Section 6-b) **– NOT COMPLIED -**
